# Parents' perception and willingness to maintain provider care continuity for their children under universal health coverage

**DOI:** 10.3934/publichealth.2019.2.121

**Published:** 2019-04-15

**Authors:** Yu Chen Tseng, I Jen Wang, Christy Pu

**Affiliations:** 1 Department of Public Health, National Yang-Ming University, Taipei 112, Taiwan; 2 Department of Pediatrics, Taipei Hospital, Ministry of Health and Welfare, Taiwan; 3 School of Medicine, National Yang-Ming University, Taipei 112, Taiwan; 4 College of Public Health, China Medical University, Taichung 40402, Taiwan; 5 College of Public Health, National Taiwan University, Taipei 10055, Taiwan

**Keywords:** continuity of care, pediatrics, contingent valuation, parents, national health insurance

## Abstract

**Background:**

Provider continuity of care (COC) is closely related to patient outcome in pediatrics. However, no study has investigated how parents perceive the importance of COC and whether their perceptions affect their willingness to make effort to maintain good provider COC for their children under universal health coverage.

**Methods:**

A cross-sectional survey was conducted between August 2017 and February 2018 across 6 different practices: 2 medical centers, 2 regional hospitals, 1 district hospital, and 1 clinic (n = 825). Parents' and caregivers' perceptions and perceived value of COC were evaluated using 7 items. The contingent valuation method was used to estimate willingness to pay and spend time.

**Results:**

Of all respondents, only 47% (n = 394) were willing to spend >30 minutes to have their children see the regular physician if the regular physician relocated. Approximately 38% (n = 302) respondents were willing to pay more than New Taiwan Dollar (NT$) 300 per month to maintain provider COC. The perception that high COC is important was associated with willingness to spend more time for maintaining high provider COC.

**Conclusion:**

Parents' perception of COC does not affect their willingness to pay for maintaining high provider COC for their children but affects their willingness to spend more time to maintain COC.

## 1. Introduction

The effect of continuity of care (COC) on patient outcome has been extensively studied in the past decade. COC can be described as the affiliation between patients and the health care system [1]–[3]. Higher COC is associated with better adherence to medication and treatment plan by patients and the consequent improvement in patient outcomes and satisfaction [4]. Moreover, COC is correlated with decreased use of emergency medical services and hospital admissions, which further decreases disease burden and cost [4]–[6].

Health care for children is different from that for adults; in that, parents or main caregivers, instead of the patient, are often the decision makers. The positive effect of high-quality COC has been shown in pediatric patients with asthma [7] or with oncology [8] and in pediatric patients admitted to intensive care units [9]. COC is also associated with decreased subsequent emergency department (ED) utilization during infancy, and thus, interventions aimed at increasing the continuity of early well-child visits may reduce ED utilization during infancy [10].

Although studies have investigated the importance of high COC in pediatric patients, those investigating how parents perceive the importance of high-quality COC and whether parents are willing to make effort to maintain good COC for their children are lacking. In addition, no studies have investigated this issue under a universal health coverage system. Universal health coverage is aimed to remove financial barriers for seeking medical care. However, efforts from the parent-side are required to maintain provider continuity. For example, parents may need to spend extra time and transportation expenses to be able to maintain good relational continuity with a physician.

The Taiwan National Health Insurance (NHI) program is a public insurance system with compulsory enrollment of all residents in Taiwan. All essential outpatient, inpatient, and pharmaceutical expenses are covered. Patients only have to pay a minimal registration fee and copayment. In addition, all children under 3 years are exempted from copayments. Patients can choose any provider at any level without referral.

COC can be categorized into 3 dimensions: information, management, and provider COC[11]. Information COC is the foundation of COC that provides for a system that can maintain and transfer patient information and preferences between health providers. Management COC entails cooperation among several providers in managing and providing patients with a comprehensive treatment plan for diseases. Provider COC refers to the degree of relationship between patients and providers[2],[3]. Several studies have indicated provider COC as a core value of primary care[12]. Patients usually prefer having a regular, primary doctor. Provider COC is particularly valued by specific population groups, such as elderly patients, young parents, and less-educated or low-income people [13]. Patients with chronic disease, psychiatric conditions, or severe illness are more likely to value provider COC than those without [13]–[15]. Provider COC has been commonly referred to as the degree to which patient care is concentrated around a single physician. Contingent to a patient–provider relationship being established, patients are more willing to express their concerns to and place trust in their regular physicians [16].

Our study contributes to the existing literature in 2 ways: First, we assessed whether parents' perception of COC under a universal health coverage system. Second, we assessed whether parents are willing to make effort to maintain good COC using the contingent valuation (CV) method, and whether they are willing to pay for high-quality COC for their children.

## 2. Methods

### 2.1. Study design and sample collection

We conducted a cross-sectional survey between August 2017 and February 2018. The survey was designed by the authors and was sent to 6 public health experts working in the academic field for validation (professors and associate professors). The questionnaire was then pretested on 30 respondents. Feedback was then sent back to the experts for discussion before the final questionnaire was formalized.

**Figure 1.**
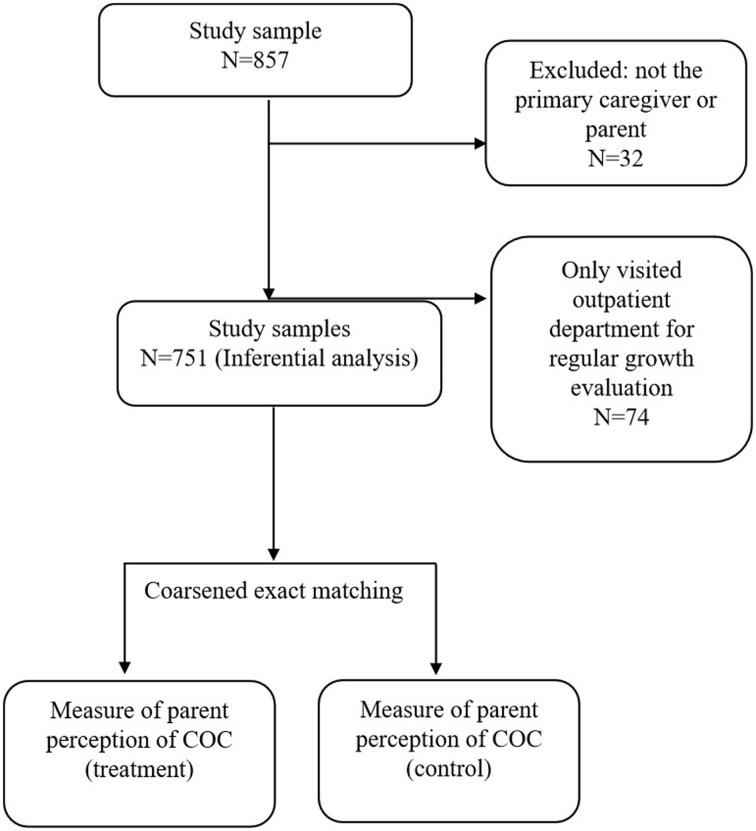
Flow chart for sample selection and analysis.

Study participants were pediatrics patients' main caregivers across 6 different practices: 2 medical centers, 2 regional hospitals, 1 district hospital, and 1 clinic. The questionnaire was self-administered and was completed on site. Every participant on average spent 10–15 minutes to complete the questionnaire. A trained staff was available on each study site to answer any question from the participant. Participants were included if they were aged ≥20 years and could communicate in Mandarin. In total, 857 respondents completed the questionnaires from outpatient departments. Rejection rate was <5%. We excluded respondents who were not parents or primary caregivers of a pediatric patient because they may not be familiar with the patient's lifestyle and health care regime. After exclusion, 825 people were included (Figure 1). Ethical approval was obtained from Taipei Hospital, Ministry of Health and Welfare, Institutional Review Board on Humanities & Social Science Research.

### 2.2. Survey measures and instrument

A series of closed-ended questions were posed in the survey. Perceptions and understanding of aspects of parents' or caregivers' COC were evaluated using 7 items. The level of provider COC was measured using a self-reported question on the frequency of changing pediatric physicians for a disease episode: “During the past year, how often had you changed pediatric physicians for the same disease?” To analyze the interpersonal trust on doctors and importance of COC per caregivers' perception, the following question was asked: “Whether you have changed the pediatric physicians or not, do you think changing physician would affect your child's therapy outcome?” (Yes, it would have a negative effect/No, it would not affect therapeutic outcome/Yes, it would have a positive effect). This question was designed to match the measure of the COC index, which has often been used to measure the degree of provider COC [17]–[19]. Given that parents may change pediatric physicians owing to circumstances that they perceive to be necessary (such as poor treatment outcome from the previous physician), we also asked the question “Do you think having a main primary pediatric physician for your child is important?” (Unimportant/Somewhat important/Very important) to see whether they would prefer no such changes if possible. We also assessed parents' trust in their physicians by asking them to score their confidence on the pediatric physicians they visited during the past year (5-point Likert scale, 1 = no trust, 5 = highly trusted).

The CV method has often been used to estimate willingness to pay in medical care studies [20]. Willingness to spend time to maintain provider COC was assessed using the question: “If the physicians your child usually seeks relocates, such that it will take longer time to reach him/her, would you be willing to spend more than 30 minutes for your child to see this physician?” Thirty minutes was chosen to be the cutoff point to better suit the local situation [21]. A similar question was asked for the maximum amount of money (in New Taiwan Dollars [NT$]) the respondent was willing to pay per month to maintain COC with the same pediatric physician (NT$0, NT$1–NT$100, NT$101–NT$300, NT$301–NT$600, NT$601–NT$1,000, and >NT$1000). NT$1000 represents about twice the amount of copayment required under the National Health Insurance program for an outpatient visit in medical centers in Taiwan.

### 2.3. Other variables

Parents or caregivers and children's covariates were collected using the self-administered questionnaire. A parent or caregiver's demographic characteristics included their relationship with the child (parent and others), age, sex, marital status (married and others), education level (holding a college degree or not) and household income per month (<NT$40,000, NT$40,000–NT$70,000, NT$70,000–NT$100,000, >NT$100,000). The children's covariates included age, sex, parents' rating of child's health (good, fair, and bad), and whether the child has severe illness. The 6 sites for sample collection were also included in the analysis. We also asked the question “During the past year, what's the reason for you to change pediatricians for your child?” Multiple responses were allowed.

### 2.4. Statistical analysis

Poststratification weighting adjustment was used to calculate population estimates representative of pediatric patients in Taiwan. Poststratification is a common technique in survey analyses for estimating population distribution in the context of survey sampling [22]. To adjust the inequal probabilities of selection into the sample for population units of analysis [23], our study samples were weighted with poststratification adjustment according to age and sex based on the 2016 annual report released by the Ministry of Health and Welfare in Taiwan. Univariate descriptive statistics were constructed to represent the demographic characteristics of the whole sample.

Because COC is more applicable to disease outcome, we excluded participants who responded that their children only had visited the outpatient department for regular growth evaluation (n = 74) to conduct inference analysis. We then used coarsened exact matching to determine the effect of each parent perception measure on willingness to pay and willingness to maintain good COC. Samples were matched using coarsened exact matching method to match the 2 groups' demographic characteristics (caregiver's sex, age, education, household income, and children's age and sex) repeatedly for each “treatment” variable. Each measure of parent perception was considered an independent “treatment” variable, and thus we repeated the entire matching process and the subsequent analysis for each measure of perception. We grouped perceptions in each item into dichotomous or trichotomous groups depending on the distribution of responses (e.g., some response categories were too small to be considered a group). We assessed the association between willingness to pay and a parent's perception of COC using multiple logistic regression models with matched and weighted sampling.

Several sensitivity analyses were performed to assess the robustness of our findings. First, we included all missing samples and used simple imputation for missing values. Second, the ordinal models for willingness to pay were used by maintaining the original response categories. Third, we included those parents who indicated they were only visiting for growth evaluation of their child. The conclusions of these analyses were similar and are available on request. All analyses were conducted using STATA (version 15.0; College Station, TX).

## 3. Results

### 3.1. Demographic characteristics

Demographic characteristics of the 825 participants included in the study are summarized in Table 1. Most participants were female (77.9%, n = 646) and married (91.2%, n = 763); more than half of those had a college degree (51.5%, n = 490). The median monthly household income was about NT$40,000–NT$70,000.

### 3.2. Question responses

Details on parents' perception and understanding of aspects of COC are summarized in Table 2. Of 825 participants, 81% (n = 700) of the respondents perceived having a single primary pediatric physician is very important, most had a confidence score of >4 for their physicians (maximum = 5); 87% (n = 713) stated that they seldom (n = 500) or never (n = 213) changed their children's physician. Regarding parents or caregivers' perceptions, <20% (n = 148) considered that changing pediatric physicians would have negative effect on therapeutic outcome of their children, and 53% respondents considered it would not affect the therapeutic outcome.

**Figure 2.**
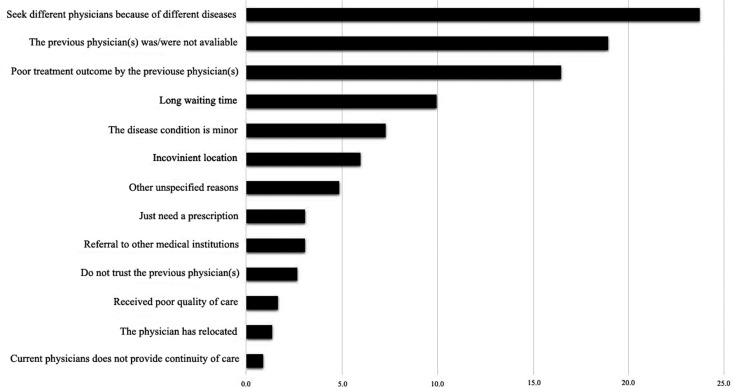
Self-reported reasons for changing pediatric physicians.

When respondents who indicated having changed pediatric physicians before (n = 604) were asked about barriers to maintaining good provider COC, most respondents responded with “Prefer to choose different physicians for different diseases” (23.7%, n = 262), followed by “The regularly visited physician is unavailable” (18.9%, n = 209) and “Current physician provides poor treatment outcome” (16.5%, n = 182). The relative percentages of reasons for a change in physician are listed in Figure 2.

**Table 1.** Participant characteristics^†^.

Characteristics	All participants (N = 825)	
Caregiver/parent	N	(%)
Parent	769	(95.2)
Age (y); mean (SD)	42	(0.3)
Female sex	646	(77.9)
College degree	490	(51.5)
Monthly household income (NT$)^††^		
<40,000	209	(26.6)
40,000–70,000	311	(36.7)
70,000–100,000	175	(21.7)
>100,000	121	(15.0)
Married	763	(91.2)
Children		
Age (y); mean (SD)	9	(0.1)
Female sex	345	(48.2)
With severe illness	35	(4.7)
Caregiver rated child's health status		
Good	266	(29.4)
Fair	440	(56.3)
Poor	155	(14.3)
Sites of sample collection		
Site no.1	312	(47.6)
Site no.2	95	(10.5)
Site no.3	178	(14.4)
Site no.4	88	(12.4)
Site no.5	102	(7.7)
Site no.6	50	(7.4)

^†^ Data presented as number (weighting percentage) of patients unless otherwise indicated. ^††^ US$1 ≈ NT$30.

### 3.3. Parents perception of care COC and willingness to spend time or money

Only 47% (n = 394) of the respondents were willing to spend >30 minutes to have their children see the regular physician if the regular physician had to relocate. Approximately 38% (n = 302) of the respondents were willing to pay >NT$300 per month (Table 2).

Parents' trust toward the pediatric physician and their perception of whether a change in pediatric physician would affect outcome were associated with willingness to spend time or money (Table 3). Male and college-educated respondents were both willing to pay and spend time. Moreover, self-reported provider COC and had higher monthly income were correlated with willingness to pay extra money. Differences in willingness to spend additional 30 minutes toward maintain COC were seen among participants from different sites.

We then used matched samples to perform multivariable regression analysis. Each perception represented a “treatment” and matching was performed for each perception, with each row representing an independent regression. The results showed that provider COC level, parent's perception of importance of COC, and parent's trust in physicians were associated with willingness to pay >NT$300 per month for maintaining provider COC (Table 4). There was no evidence of parent's perception significantly affecting their willingness to spend time. In all models, respondents' household income was strongly correlated with willingness to pay money.

**Table 2.** Parents' perception of COC for their children.

Item descriptions		All participants
Parent's perception of continuity		n = 825
During the past year, how often have you changed pediatric physicians for the same disease?	Never	213 (33.9)
	Seldom	500 (53.0)
	Often	32 (3.2)
	Always	4 (0.3)
	Growth-evaluation only	74 (9.4)
	No response	2 (0.2)
Whether you have changed the pediatric physicians or not, do you think that changing physicians affect your child's therapeutic outcomes?	It would have a negative effect	148 (18.1)
	It would not affect outcome	426 (52.9)
	It would have a positive effect	245 (27.8)
	No response	6 (1.2)
Do you think having a primary pediatric physician is important for your child?	Unimportant	9 (1.7)
	Somewhat	114 (17.2)
	Very important	700 (81.0)
	No response	2 (0.1)
Score a overall confidence level for all the pediatric physicians you have met during the past year.	1 (no confidence)	3 (0.2)
	2	15 (1.2)
	3	179 (21.2)
	4	387 (45.0)
	5 (Very confident)	240 (32.3)
	No response	1 (0.1)
Value of continuity		
Would you change the pediatric physicians if he/she had relocated and you needed to spend more than 30 minutes to get there?	Yes	430 (52.7)
	No	394 (47.0)
	No response	3 (0.3)
How much money you would pay per month to maintain your child's regular pediatric physician if he/she had relocated?	NT 0	46 (5.0)
	NT 1∼100	152 (19.1)
	NT 101∼300	320 (38.3)
	NT 301∼600	152 (17.5)
	NT 601∼1000	92 (12.2)
	>NT 1000	58 (7.4)
	No response	5 (0.5)

**Table 3.** Characteristics associated with willingness to spending time or money to maintain COC on weighted sampling.

	Will spend more than 30 min			Will pay more NT.300/month		
	%^†^	n	P value	%	n	P value
Have changed physician						
Yes	42.1	(234)	0.087	33.1	(184)	0.004
No	52.4	(115)		49.5	(94)	
Parent's perception of continuity						
Changing physician is good for therapy						
Yes	41.7	(101)	0.011	44.8	(103)	0.006
It doesn't matter	42.5	(167)		31.6	(115)	
No	62.7	(81)		52.7	(60)	
Continuity is important						
Yes	46.4	(309)	0.858	43.3	(256)	0.005
No	44.9	(42)		20.5	(22)	
Trust in physician						
Trust	49.8	(289)	0.007	43.3	(230)	0.001
Trustless	32.6	(62)		24.4	(48)	
Parents/caregivers						
Male sex	58.7	(84)	0.010	57.2	(79)	<0.001
Age						
≦ 40 y	43.7	(229)	0.413	36.1	(187)	0.259
> 40 y	48.0	(122)		41.9	(91)	
Education						
High school and bellow	53.7	(164)	0.009	33.0	(94)	0.032
College and above	39.1	(186)		44.8	(184)	
Monthly income						
<NT. 40,000	52.0	(102)	0.372	19.2	(51)	<0.001
NT. 40,000∼70,000	46.2	(127)		36.5	(91)	
NT. 70,000∼100,000	37.8	(68)		50.5	(75)	
>NT.100,000	46.7	(51)		65.6	(61)	
Children						
Male sex	47.3	(203)	0.634	39.9	(165)	0.804
Age						
≦6 y	49.6	(235)	0.242	37.5	(180)	0.563
> 6 y	43.9	(116)		40.2	(98)	
Caregiver-reported child's health status						
Good	50.1	(115)	0.067	39.8	(81)	0.556
Fair	41.2	(178)		37.2	(149)	
Poor	57.9	( 57)		45.7	(47)	
Sites of sample collection						
Site no.1	37.0	(101)	<0.001	37.2	(96)	0.230
Site no.2	74.8	(61)		52.1	(52)	
Site no.3	40.7	(67)		36.7	(51)	
Site no.4	48.1	(46)		38.1	(26)	
Site no.5	41.4	(42)		27.3	(27)	
Site no.6	71.4	(34)		51.0	(26)	

Note: ^†^ The percentage presented here is the percentage of the number of participants in the bracket. For example, 42.1% of the 234 respondents who reported “Yes” for changing their physician responded they are willing to spend 30 minutes to maintain provider COC.

**Table 4.** Parents' perceptions associated with willingness to pay to maintain COC among all participants^†^.

	Willing to spend more than 30 minutes		Willing to pay more than NTD300/month	
	Odds ratio	95% CI	Odds ratio	95% CI
Provider continuity level (ref: never changed)				
Seldom changed	0.76	0.47–1.22	0.48	0.28–0.80
Often changed	0.46	0.14–1.52	0.23	0.07–0.77
Effect of changing physicians for therapy (ref: negative)				
No effect	0.46	0.21–1.01	0.67	0.34–1.30
Positive	0.45	0.19–1.04	1.08	0.49–2.36
Continuity is important (ref: unimportant)	0.61	0.28-1.31	3.12	1.38–7.03
Trust in physician (ref: trustless)				
Trust	1.09	0.31–3.87	3.22	0.89–11.63
Fair	0.74	0.20–2.72	1.06	0.28–4.01

Note: ^†^ Each perception is considered a separate “treatment” and is estimated by separate models. All models were controlled by caregiver's age, sex, education, income, children's age, children's sex, children's health status, and site of sample collection.

## 4. Discussion

Although several studies have highlighted the importance of COC for pediatric patient outcomes, no studies have investigated how parents perceive COC, and whether parents are willing to make effort to maintain good COC under a universal coverage system. Patients consulting the same doctor are more willing to share their concerns and expectations with their medical provider, and physicians are more likely to build an accumulated knowledge about their patient's clinical condition and preference [18]. However, we found that although most parents believe having a primary pediatric physician is important, a notable number of the respondents indicated that they believed changing physicians would improve therapeutic effect.

We also attempted to capture which aspect of COC in parents' perception may affect their perceived value of provider COC. In parents' perception of COC, patient trust is an essential component. This study showed that when parents trust their children's physician, they are more willing to pay additional money to consult the same pediatric physician. This is in line with observations among adult patients. Patient trust may be fundamental to provider COC; only when patients trust their provider will they be willing to maintain the relationship.

The apparent inconsistency in parents' perception of COC and willingness to spend time and money may be due in part to a social response bias that led parents to overestimate the value of provider COC [24]. The possible explanation is the high medical accessibility and very few barriers to limit patients from changing physicians in Taiwan, with 99% people enrolled in the NHI program. Patients can choose their primary care provider freely without the need for a referral to seek consultation at a higher-level health care facility. After implementing the NHI-PharmaCloud system, patient's disease and medication history are available to all qualified providers. Patients need not maintain a regular provider or site of care to ensure COC in medical history. In this study, parents' perceived value of COC, which included patient's trust and the importance of COC, was only associated with willingness to spend additional money. A reason for this observation could be that most parents in this study were aged 30–40 years; such parents tend to be more willing to spend more money than older people to maintain provider COC. Furthermore, patients and parents would be more willing to pay extra money to avoid long waiting times.

A few studies regarding the importance of COC in adult patients have been conducted. In a population survey of 314 participants, 85% of respondents were willing to wait 3–4 days to see their family doctor [25]. In another survey, 658 Kentucky adults who indicated they usually seek care from the same physician were asked whether they would wait to see their usual physician if they had an acute non–life-threatening condition and their usual physician is unavailable; <42% respondents were willing to wait >1 day to see their family physician, but only patients with asthma were more likely to wait longer to maintain provider COC [26]. In another survey of 2500 participants, 55% respondents were willing to spend an additional US$5–US$10 per month and only 22% participants would be willing to drive >60 minutes to maintain COC with their primary care physicians [24]. The less-educated and low-income patients were more willing to drive longer rather than pay more to maintain COC [24].

Provider (physician or practice) characteristics affect provider COC [27]. However, definitive evidence on this association is lacking. In our survey, participants from each site were included from 1 or more physicians' outpatient clinics. This implies that participants enrolled from a single practice might have been consulted by different physicians and might have had varying experiences, which might have affected their willingness to pay for provider COC. In general, patients more likely value COC when they are satisfied with the service and perceive that their physician completely understands their condition [15]. The provider and practice characteristics might have significantly affected our analysis, a plausible reason for the nonsignificant association between asthma and parents' willingness to maintain provider COC.

Some recent studies have debated on the necessity of provider COC: Arguments have been made against forced patient–physician COC by government policies. Patients should have the freedom to select or change their physicians should they prefer COC [28]. In patients with chronic conditions, management transforms to multidisciplinary team care, with the health care system paying less attention to ensuring provider COC [29]. Pediatric patients are more vulnerable; hence, focusing primarily on parents' attitude toward COC among the pediatric population is crucial. The demand for health care among children depends on their parents or main caregivers. Although provider COC is a much simple, effective method to improve a child's health status, parents may underestimate or misunderstand the importance of COC for their children. The policymakers and the health care system should not only promote the benefits of COC among patients but also make effort to remove barriers to establishing COC between patients and providers.

This study has some limitations. First, the findings of this study cannot be generalized to all patient populations, given that this survey included only patients at outpatient departments of hospitals and clinics. Moreover, most participants were enrolled from urban areas and hence our findings may not be applicable to patients from rural areas. Although we attempted adjustment for unequal probability sampling using poststratification weighting, enrollment was nonrandom, precluding the study population from being representative of all patient population. Second, the measures of variables may not have been precise given they were obtained using self-administered questionnaires. Recall bias might have been present in some responses. While caregivers declared that they never or seldom changed their children's physicians, they might have been overconservative in estimating the frequency of changing physicians. However, this is not unique in survey studies.

## 5. Conclusions

Willingness to maintain high COC are affected by factors other than financial barriers to medical utilization under a universal coverage system. Although provider COC is highly associated with good patient outcome, parents may not always believe maintaining high provider COC is necessary for their child's therapeutic outcome. This perception may negatively affect their medical decisions for their children. Policies should aim at educating parents on the importance of maintaining high provider COC for their children.
